# Emerging Frontiers in acute kidney injury: The role of extracellular vesicles^☆^

**DOI:** 10.1016/j.bioactmat.2025.02.018

**Published:** 2025-02-18

**Authors:** Sirui Li, Lan Zhou, Yu Huang, Shupei Tang

**Affiliations:** a Institute of Immunology, Third Military Medical University, Chongqing, 400038, China; b Department of Urology, 79th Military Group Hospital of the Chinese People's Liberation Army, Liaoyang, Liaoning, 111000, China; c Frontier Medical Training Brigade, Third Military Medical University, Xinjiang, 831200, China; d Department of Gastroenterology, Xinqiao Hospital, Third Military Medical University, Chongqing, 400037, China; e Department of Emergency Medicine, Shigatse Branch, Xinqiao Hospital, Third Military Medical University, Shigatse, 857000, China

**Keywords:** Acute kidney injury, Extracellular vesicles, Biomarkers of AKI diagnosis, Engineered or modified EVs

## Abstract

Acute kidney injury (AKI) remains a prevalent and critical clinical condition. Although considerable advancements have been achieved in clinical and fundamental research in recent decades, the enhancements in AKI diagnosis and therapeutic approaches, such as the development of emerging biomarkers including neutrophil gelatinase-associated lipocalin (NGAL) and liver fatty acid-binding protein (FABP1) for early detection of AKI and the exploration of “goal-directed" hemodynamic treatment methods and renal replacement therapies, have yet to fulfill the demands of modern medicine. Extracellular vesicles (EVs) serve as pivotal messengers in cell-to-cell communication, exerting a vital impact on both physiological and pathological processes. They exhibit immense potential as disease regulators, innovative biomarkers, therapeutic agents, and drug delivery vehicles. In recent times, the diagnostic and therapeutic potential of EVs in AKI has garnered widespread recognition and exploration, making them a focal point in clinical research. Consequently, a comprehensive overview of EVs' role in AKI is of great importance. This review delves into the multifaceted roles of EVs from diverse cellular sources, including tubular epithelial cells (TECs), mesenchymal stem cells (MSCs), progenitor cells, platelets and macrophages, within the context of AKI. It scrutinizes their contributions to disease progression and mitigation, their diagnostic marker potential, and encompasses a variety of conventional and novel EVs extraction techniques suitable for AKI clinical applications. Moreover, it underscores four innovative strategies for engineering EVs to boost production efficiency, targeting precision, circulatory stability and therapeutic potency. These advancements pave the way for novel approaches in the diagnosis and treatment of AKI. We are optimistic that as research into EVs progresses, the future will bring about earlier detection, more tailored treatments, and a more holistic management of AKI.

## Highlights

• Summarized key insights on multi-source EVs in AKI mechanisms and potential therapies.
• Reviewed the potential of blood and urine EVs as biomarkers for AKI.
• Analyzed traditional and emerging EV isolation techniques, comparing those for AKI translation.
• Investigated methods to enhance engineered EVs for AKI therapy.

## Graphical abstract

**Figure:**
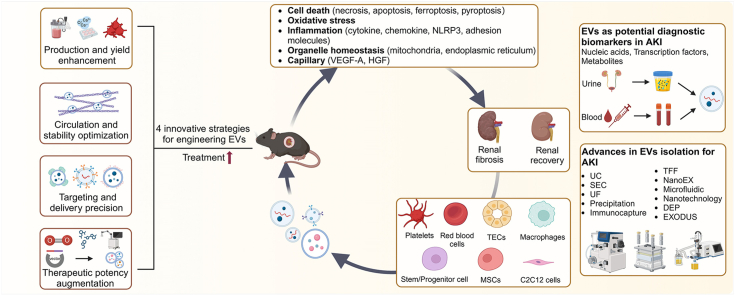

## 1 Introduction

Acute kidney injury (AKI) is a prevalent critical condition, with an estimated 13.3 million cases diagnosed globally each year, resulting in approximately 1.7 million fatalities [[1], [2], [3]]. Characterized by a sudden decrease in kidney function within 1–7 days or persistent renal insufficiency beyond 24 h, AKI presents with elevated serum creatinine (Scr), azotemia, and electrolyte imbalances, often accompanied by oliguria (<400ml/24 h or <17 ml/h) or anuria (<100ml/24 h) [4,5]. More gravely, 30–70 % of AKI patients evolve into chronic kidney disease (CKD), potentially leading to end-stage renal disease [6]. Hospitalized patients, particularly those in intensive care units, have a 10–15 % incidence of AKI, with rates exceeding 50 % in critical settings [1]. AKI can be categorized based on etiology into pre-renal (due to conditions like surgery, trauma, burns, and sepsis that reduce renal perfusion), intrinsic renal (caused by direct renal parenchymal or vascular injury from nephrotoxic agents or sepsis), and post-renal (related to increased intrarenal pressure from urinary tract obstruction) factors [7]. The diagnosis of AKI relies primarily on clinical presentation and assessment of renal function (serum concentration of Scr and blood urea nitrogen (BUN)), with renal biopsy as the definitive method for evaluating renal damage. Thus, the development of novel biomarkers for diagnosing and monitoring AKI pathophysiology is crucial [8]. In addition, management of AKI is currently centered around cause removal and supportive care, lacking a specific therapeutic intervention [5]. The pursuit of innovative treatment strategies for AKI holds significant clinical importance.

**Fig. 1:**
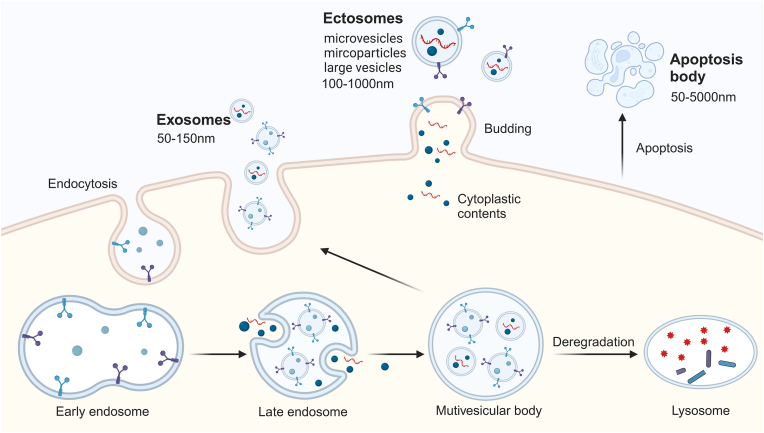
EVs can be primarily categorized into three types based on their origin: exosomes, ectosomes, and apoptosis bodies.

In line with the Minimal Information for Studies of Extracellular Vesicles (MISEV) guidelines from 2018 to 2023, extracellular vesicles (EVs) are defined as cell-derived particles enclosed by a lipid bilayer, lacking the ability to replicate independently due to the absence of a functional nucleus [9,10]. Categorized by their cellular origins, EVs are primarily divided into exosomes, ectosomes and apoptotic bodies (Fig. 1). These vesicles are enriched with proteins, nucleic acids, lipids, and other bioactive molecules, facilitating cellular communication and engagement with the extracellular milieu [11]. Studies have highlighted the pivotal roles of EVs in inflammation, immune regulation, and tissue repair [12]. Given the lack of consensus on specific markers for EV subtypes, MISEV advocates for categorizing EVs subpopulations based on their physicochemical properties, biochemical content, or cellular source, rather than labeling them as exosomes or microvesicles. For example, those under 200 nm are designated as small EVs (sEVs), while those above 200 nm are known as large EVs (lEVs) [9,10]. With deepening research, EVs, attributed to their low immunogenicity and favorable tissue compatibility, have become pivotal in intercellular signaling, playing significant roles in a spectrum of diseases, including renal pathologies [13], and are increasingly applied in regenerative medicine, nanomedicine, and as diagnostic biomarkers [[14], [15], [16]].

Functional proteins and nucleic acids within EVs can modulate numerous post-AKI renal processes, including cell proliferation, apoptosis, inflammation, and oxidative stress, either directly or indirectly. The role of EVs from different cell types—like mesenchymal stem cells (MSCs) and progenitor cells—in AKI is also gaining attention and can lead to new treatment options. Additionally, EVs in the urine and blood of AKI patients show marked changes in biomarker profiles early in the disease course, outperforming traditional Scr and BUN markers in sensitivity, indicating their potential for early diagnosis and disease monitoring. Recognizing the pivotal role of EVs in the treatment of various diseases, researchers have developed a suite of extraction technologies and engineered modifications aimed at enhancing EVs' production yield, circulatory stability, targeting precision, and therapeutic potency, thereby enabling more effective disease treatment, including AKI intervention. This review begins by summarizing the functions and mechanisms of EVs from various cellular origins in AKI, followed by the progress using EVs and their contents as early diagnostic markers for AKI. Subsequently, it introduced a range of innovative and traditional EVs extraction techniques, and specifically discussed the applicable methods in response to the clinical application needs of AKI. Finally, it delves into four strategies for engineering EVs to augment treatment efficacy in AKI. By consolidating this information, we hope to provide novel insights for AKI management, especially regarding early detection, personalized treatment plans, and the innovation of pharmaceuticals and therapeutic strategies.

## 2 The functions and therapeutic applications of EVs in AKI

**Fig. 2:**
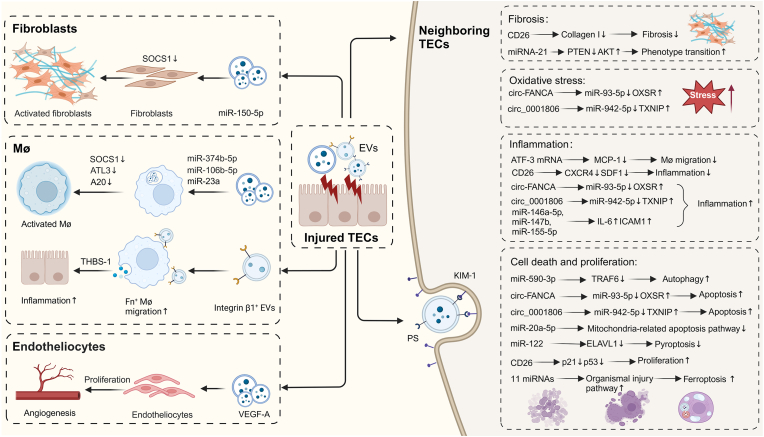
The dual role of TECs-derived EVs in the progression and alleviation of AKI. Upon the onset of AKI, compromised TECs emit a substantial quantity of EVs that modulate the progression of AKI through intercellular signaling. These EVs can interact with KIM-1 on the TECs surface via PS, gaining entry into the cells. They contribute to the exacerbation of AKI by fueling inflammation, oxidative stress, cell death, and fibrosis. Moreover, EVs have the capacity to infiltrate fibroblasts, reducing the expression of SOCS1, which amplifies kidney fibrosis, or they can enter and activate macrophages, intensifying inflammatory responses. However, on the other hand, EVs that enter TECs contain ATF-3 mRNA and CD26, which can dampen inflammatory reactions, while miR-122 can inhibit pyroptosis, thereby mitigating TECs damage. Additionally, EVs that infiltrate vascular endothelial cells carry VEGF-A, promoting angiogenesis and reducing tissue damage.

In recent years, the role of EVs from various cellular origins in the progression and therapeutic applications of AKI has been a subject of intensive research. Current studies are particularly focusing on the impact of tubular epithelial cells (TECs)-derived EVs on AKI progression or alleviation and the potential of MSCs and stem/progenitor cells-derived EVs in AKI treatment. The importance of synthesizing and summarizing these research advancements lies in their potential to revolutionize our understanding and management of AKI, highlighting the need for further investigation into these promising cellular communicators. In this review, while some sections use “EVs" as stated, other literature we consulted refers to them as exosomes or ectosomes (microvesicles/microparticles). To clarify subtype distinctions, we adhere to the MISEV2018 and MISEV2023 guidelines, classifying EVs from related studies into sEVs and lEVs based on their average size (Fig. 2, Table 1).

**Table 1:** The role of TECs-derived EVs in AKI progression and alleviation.

Condition	Model	Isolation	Cargo	Mechanism	Outcome	Ref
IR	Mouse, mTECs	UC	PS	PS/KIM-1-mediated EVs uptake by TECs	promote inflammation	[24]
	Mouse, mTECs	UC	miR-374 b-5p	SOCS1↓	promote inflammation and M1 polarization	[28]
	Mouse, mTECs, HK‐2	UC	miR‐106 b‐5p	ATL3↓	transit ER stress, promote M1 polarization	[30]
	Mouse, HK‐2	UC + UF	integrin β1	integrin β1/THBS-1 interaction, THBS-1/CD36 interaction	promote Mø infiltration and inflammation	[31]
	Mouse, HK‐2	GC + UF	miR-20a-5p	mitochondria/apoptosis pathway↓	promote TECs proliferation, improve mitochondrial functions	[35]
	Mouse, HK‐2	UC	VEGF-A	VEGF-A signaling↑	promote angiogenesis	[38]
	Mouse, HK‐2	UC	CD26	p21↓p53↓SDF1↓ CXCR4↓ collagen I↓	promote proliferation, anti-inflammation, anti-fibrosis	[39]
	hPTCs	UF + SEC + UC	11 varieties of miRNA	Organismal injury pathway↑	promote ferroptosis and fibrosis	[25]
	Mouse, NRK-52 E	PPD	miR-150–5p	SOCS1↓	promote fibrosis	[29]
	Mouse, NRK-52 E	UC	ATF3 RNA	MCP-1↓	reduce Mø migration	[34]
	HK-2	Isolation Kit	miR-590–3p	TRAF6↓	promote autophagy	[36]
LPS	HK2	Isolation Reagent	circ-FANCA	miR-93–5p↓OXSR↑	promote apoptosis, inflammation and oxidative stress	[21]
	HK2	UF + Isolation reagent	circ_0001806	miR-942–5p↓TXNIP↑	promote apoptosis, inflammation and oxidative stress	[22]
UUO	Mouse, NRK-52 E	UC	miRNA-21	PTEN↓AKT↑	phenotype transition	[26]
IR, UUO	Mouse, mTECs	UC	miRNA-23a	HIF-1 upregulates miRNA-23a, A20↓	promote inflammation	[27]
Cisp	Mouse, HK-2	UC	miRNA-122	ELAVL1↓	*anti*-pyroptosis	[37]
IFN-γ, TNF-α, IL-1β	hPTCs	SEC	miR-146a-5p, miR-147 b, miR-155–5p	IL-6↑ICAM1↑	promote inflammation	[18]

### 2.1 TECs-derived EVs in AKI progression and alleviation

In AKI patients, the injury and death of TECs are central histological changes that precipitate the decline in renal function. TECs damage, which can stem from sepsis, ischemia-reperfusion (IR) injury, drug toxicity, and other factors, can directly trigger AKI. As this damage intensifies, it may result in the loss of epithelial cells, tubular fibrosis, tubular cell atrophy, and interstitial inflammation and fibrosis, potentially advancing AKI to CKD [17]. Consequently, TECs have a pivotal role in both the initiation and progression of kidney diseases. They can act protectively but also exacerbate AKI through EV-mediated communication with other cells, embodying a double-edged sword.

Sepsis, a leading cause of AKI, triggers a cascade of complex mechanisms including inflammation, oxidative stress, and metabolic reprogramming. Injured TECs worsen or mitigate AKI progression by secreting EVs that act through multiple pathways. Under septic conditions, the levels of miR-146a-5p, miR-147 b, and miR-155–5p in the EVs derived from TECs were substantially upregulated. These microRNAs were functionally involved in the regulation of the TLR signaling pathway and inflammatory mediators, including IL-6 [18]. Beyond miRNAs, circular RNAs (circRNAs), characterized by their closed-loop structure, are non-coding RNAs intimately linked to a spectrum of human diseases [19]. Within the context of sepsis-related AKI, both in patients and in LPS-stimulated HK-2 cells, circVMA21 is shown to bind directly to miR-9-3p, leading to the upregulation of SMG1 and the consequent mitigation of inflammation and apoptosis [20]. Conversely, the overexpression of circ-FANCA directly interacts with miR-93–5p, enhancing the expression of oxidative stress responsive 1 (OXSR1), which suppresses cellular vitality and amplifies apoptosis, inflammation, and oxidative stress [21]. Furthermore, the upregulation of Circ_0001806 [22] and Circ_0001818 [23] targets miR-942–5p and miR-136–5p, respectively, modulating downstream TXNIP molecules and exacerbating renal injury associated with sepsis. These circRNAs are found within sEVs derived from TECs, highlighting TECs' role in AKI. They also hold promise as potential therapeutic targets and as valuable diagnostic indicators.

IR-AKI is characterized by tissue hypoxia and ischemia, researchers have focused on sEVs from damaged TECs, which have a key role in cell communication and are significant drivers of AKI. Injured TECs expressed the kidney injury molecule-1 (KIM-1) protein, whose ligand, phosphatidylserine (PS), was present in sEVs secreted by the damaged TECs. Therefore, sEVs could be taken up by other TECs that highly expressed KIM-1, thereby exacerbating tubular cell inflammation and promoting renal interstitial fibrosis [24]. Recent intriguing discoveries have illuminated the mechanisms by which sEVs function upon entering cells: under hypoxic stimulation, TECs produced sEVs carrying at least 11 types of RNA messages associated with tissue injury. These sEVs could even trigger ferroptosis in normal TECs, demonstrating that damage signals could propagate from injured TECs to neighboring cells. The phenomenon of ferroptosis spreading among TECs was confirmed in patients with the transition from AKI to CKD through renal biopsies and *in vitro* experiments with urinary sEVs, and it could be ameliorated with RNase and ferroptosis inhibitors [25]. Nevertheless, the precise pathways through which EVs communicate among TECs, and whether they instigate other forms of cell death aside from ferroptosis, are questions that demand deeper investigation. The underlying mechanisms of action also necessitate further experimental and analytical elucidation. For example, lEVs shed by compromised TECs, enriched with miR-21, are transmitted to neighboring TECs via a paracrine mechanism. This interaction suppresses PTEN protein, thereby activating the AKT signaling pathway and enhancing fibrosis post-renal injury [26]. Furthermore, sEVs, through their cargo of RNAs, intensify inflammatory responses and organelle stress, thus facilitating the advancement of AKI and its transition to CKD. HIF-1α stimulates the production of sEVs from TECs that are rich in miRNA-23a. Upon macrophage uptake, these sEVs suppress A20, tipping the balance towards a pro-inflammatory phenotype and intensifying interstitial inflammation. Notably, sequestering miRNA-23a within sEVs markedly reduces this inflammation [27]. Similarly, miR-374 b-5p, found in sEVs after IR, targets SOCS1 in macrophage absorption, driving them towards an M1 phenotype and worsening renal inflammation [28]. Moreover, miR-150-5p-laden sEVs, when internalized by fibroblasts, also activate these cells via the SOCS1 pathway, hastening the fibrotic response in AKI [29]. IR also triggers endoplasmic reticulum stress in TECs, and this perilous signal, borne by sEVs harboring miR-106 b-5p, may incite macrophage M1 polarization via the ATL3 pathway, further fueling renal inflammation [30]. Moreover, injured TECs produce a plethora of chemokines and integrin β1-rich EVs that recruit a large number of mononuclear phagocytes expressing pro-inflammatory cytokines and thrombospondin-1 (THBS-1). These mononuclear phagocytes, relying on the interaction of integrin β1 and THBS-1, lead to further infiltration of inflammatory cells [31]. Therefore, targeting the within these sEVs from compromised TECs presents a promising therapeutic avenue for mitigating renal inflammation and fibrosis. It is also notable that miR-687, contained in sEVs released by injured TECs into circulation, can induce distant hepatic injury, and which can be attenuated by Thymoquinone [32]. This interplay underscores the renal system's interconnectedness with other organs through sEVs' communication, with the potential to propagate injury signals to distant sites. It highlights the therapeutic potential of sEVs in treating AKI and forestalling injury in other organs. Collectively, these insights highlight the pivotal role of TECs in AKI pathogenesis, with their EVs serving as messengers to distant organs and interacting with neighboring TECs. This dual role could be harnessed through sEVs engineering or modification to favor therapeutic interventions in AKI.

It is worth noting that EVs originating from TECs are not just contributors to AKI progression, a wealth of research has demonstrated their important role in countering AKI-induced damage and fostering kidney repair. For example, a previous study has demonstrated that the intravenous injection of EVs from healthy adult rat TECs into experimental rats with AKI significantly reduced TECs injury, neutrophil infiltration, renal fibrosis, and vascular loss [33]. In contrast, injured TECs exhibit more complex defense mechanisms. sEVs derived from injured TECs are enriched with ATF3 RNA, which downregulates MCP-1 expression in TECs, curtailing macrophage infiltration and reducing inflammation [34]. MiR-20a-5p, contained within these sEVs, hinders acute tubular damage by suppressing mitochondrial damage and apoptosis in TECs [35]. In cardiopulmonary bypass surgery, where systemic IR is common and the kidneys are particularly vulnerable, sEVs from damaged TECs containing miR-590–3p can initiate autophagy in TECs by targeting TRAF6, ultimately improving AKI outcomes [36]. It is worth mentioning that cisplatin, which induces acute TECs injury, triggers the release of miR-122-enriched sEVs that modulate embryonic lethal abnormal vision (ELAVL1) expression, thereby inhibiting pyroptosis to exert a protective effect [37]. In addition to RNAs, they also harbor vascular endothelial growth factor-A (VEGF-A), which targets peritubular capillary endothelial cells, enhances vascular permeability, and sustains capillary density by promoting their proliferation, thereby to some extent, preventing the transition from AKI to CKD [38]. Furthermore, CD26-positive sEVs downregulate the expression of p53 and p21, thereby preventing cell cycle arrest. They also diminish the expression of SDF1, a downstream molecule of CXCR4, which curbs inflammatory infiltration and restricts excessive collagen I synthesis, consequently alleviating renal fibrosis [39].

To sum up, extensive TECs death and renal interstitial fibrosis are the pivotal pathophysiological alterations that drive the progression from AKI to irreversible CKD in patients. Research suggests that danger signals, including inflammation, hypoxia, and drug exposure, not only inflict injury on TECs but also spread via EVs among these cells, fostering TECs demise, inflammatory cell infiltration, and renal fibrosis, ultimately precipitating CKD. However, the interaction between injured TECs and renal epithelial cells via EVs hints at the kidney's innate self-defense mechanisms against these danger signals. Thus, targeting the production of EVs or their specific cargo, based on the propagation of these danger signals and the defensive responses of TECs, could represent a promising therapeutic approach.

### 2.2 MSCs-derived EVs in AKI treatment

**Fig. 3:**
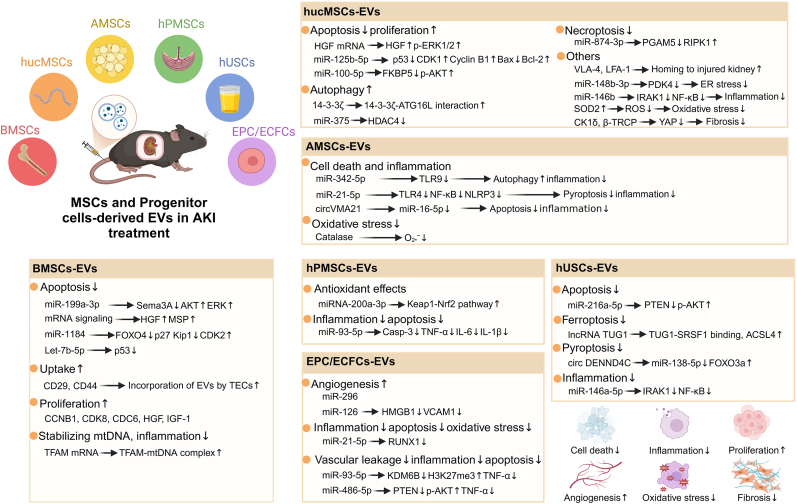
The potential roles of MSCs and progenitor cell-derived EVs in the treatment of AKI. In the realm of regenerative medicine, the significance of diverse MSCs and progenitor cells is escalating, especially regarding their secreted EVs which demonstrate substantial potential in AKI therapy. We detail the contributions of EVs from six extensively studied cell subtypes-BMSCs, hucMSCs, AMSCs, hPMSCs, hUSCs and EPC/ECFCs-in mitigating AKI. Evidence suggests that these EVs ameliorate AKI and hinder its transition to CKD by fostering cell proliferation and angiogenesis, curbing diverse cell death mechanisms including apoptosis, ferroptosis, pyroptosis, necrosis, and autophagy, and by dampening inflammation, oxidative stress, and fibrosis.

MSCs represent a diverse group of multipotent stem cells that can be sourced and differentiated from a range of tissues, such as bone marrow, adipose tissue, umbilical cord, placenta, skin, blood, urine, and even induced pluripotent stem cells (iPSCs). They have become a cornerstone in regenerative medicine, particularly noted for the paracrine effects of their EV secretions, which have an important role in tissue repair across a spectrum of diseases affecting the musculoskeletal, nervous, and urinary systems, among others [40]. Moreover, certain EVs are capable of circulating in the bloodstream, infiltrating various tissues, and even breaching the blood-brain barrier [41]. As a result, EVs have attracted considerable attention for their vast medical potential [42]. A multitude of studies underscore the importance of EVs from various origins in the trajectory of renal diseases, including both AKI and CKD, thereby showcasing substantial therapeutic promise in renal research [[43], [44], [45]]. Notably, MSCs from distinct sources display notable heterogeneity, and thus, the effector molecules they carry within their EVs also differ in their mechanisms and impacts on AKI [46,47] (Fig. 3, Table 2).

**Table 2:** The role of MSCs-derived EVs for AKI therapy.

Source	Condition	Model	Isolation	Cargo	Mechanism	Outcome	Ref
BMSCs	IR	Rat	Isolation reagent	RNA	TNF-α↓NF-κB↓IFN-γ↓IL-6↓Casp-9↓Casp-3↓Bax↓	anti-inflammation and apoptosis	[51]
	IR	Mouse	UC	miR-199a-3p	Sema3A↓AKT↑ERK↑	anti-apoptosis	[53]
	Rha	Mouse	UC	CD29, CD44, mRNA	incorporation by TECs HGF↑MSP↑	promote proliferation, anti-apoptosis	[52]
	Cisp	HK-2	UC	miR-1184	FOXO4↓p27 Kip 1↓CDK2↑	anti-apoptosis	[54]
	Cisp	Mouse, hPTCs	UF	Let-7b-5p	p53↓	anti-apoptosis	[55]
	IR	Mouse, HK-2	UC	TFAM mRNA	TFAM↑TFAM-mtDNA complex↑	stabilize mtDNA, anti-inflammation	[56]
	Cisp	Mouse, hPTCs	UC	mRNA	Bcl-xL↑Bcl2↑BIRC8↑ Casp1↓Casp8↓LTA↓	anti-apoptosis	[57]
	Glycerol	Mouse, mTECs	UC	CCNB1, CDK8, CDC6, HGF, IGF-1	/	promote proliferation, anti-apoptosis	[61]
hucMSCs	Cisp	Rat, NRK-52 E	UF + UC	/	p-p38↓ p-ERK1/2 pathway↑	promote proliferation, reduce apoptosis and oxidative stress	[65].
	IR	Rat, rTECs	UC	HGF mRNA	ERK1/2 pathway↑	promote cell dedifferentiation and growth	[66]
	IR	Mouse, HK-2, mTECs	GC + UC	VLA-4, LFA-1, miR-125 b-5p	p53↓CDK1↑ Cyclin B1↑Bax↓Bcl-2↑	anti-apoptosis	[67]
	IR	Mouse, HK-2	UC	miR-148 b-3p	PDK4↓	inhibit ER stress	[68].
	IR	Mouse, HK-2	UC	miR-100–5p	FKBP5↓p-AKT↑	anti-apoptosis	[69].
	Cisp	Rat, NRK-52 E	Isolation kit	14-3-3ζ	14-3-3ζ-ATG16L interaction↑	activate autophagy	[71]
	CLP	Mouse	UC	miR-375	HDAC4↓	promote autophagy, suppress T cell apoptosis	[72]
	IR, Cisp	NRK-52 E	UC	/	NLRP3↓GSDMD↓Casp-1 p20↓IL-1β↓	*anti*-pyroptosis	[73]
	UUO	Mouse, HK-2	UC	miR-874–3p	PGAM5↓RIPK1↑	*anti*-necroptosis,stabilize mitochondrial	[74]
	UUO	Rat, NRK-52 E	UF + UC	CK1δ, β-TRCP	YAP↓	inhibit renal fibrosis	[75]
	CLP	Mouse, HK-2	UF + UC	miR-146 b	IRAK1↓NF-κB↓	anti-inflammation	[76]
	IR	Mini pig	UF + UC	/	Klotho↑BMP-7↑NF-κB↓STAT3↓VEGF-A↑	promote regeneration and angiogenesis, anti-inflammation,	[77]
	IR	Mouse, HK-2	UC	SOD2	ROS↓	reduce oxidative stress	[78]
AMSCs	IR	Rat, HK-2	UC	Catalase	O_2_^•−^↓	reduce oxidative stress	[83]
	CLP	Mouse,	UC	/	SIRT1↑NF-κB p65↓TNF-α↓Bax↓Bcl-2↑Casp-3↓Casp-9↓	anti-inflammation and apoptosis	[84]
	LPS, CLP	Mouse, HK-2	UC	miR-342–5p	TLR9↓	enhance autophagy, reduce inflammation	[85]
	LPS	Mouse, HK-2	UC	miR-21–5p	TLR4↓NF-κB↓NLRP3↓	*anti*-pyroptosis and inflammation	[86]
	LPS	Mouse, HK-2	Isolation kit	circVMA21	miR-16–5p↓	anti-inflammation and apoptosis	[88]
hPMSCs	IR	Mouse, HK-2	UC	miRNA-200a-3p	Keap1-Nrf 2 signaling↑	antioxidant effects	[90]
	IR	Mouse	UC	miR-93–5p	Casp-3↓TNF-α↓IL-6↓IL-1β↓	anti-inflammation and apoptosis	[91]
	IR	Mouse	UC	/	Sox9^+^ cells proliferation↑	promote regeneration, anti-apoptosis	[92]
hWJMSCs	IR	Rat	UC	miR-15a, miR-15 b, miR-16, miR-195, miR-424, miR-497	CX3CL1↓	reduce inflammatory infiltration	[95]
	IR	Rat	UC	/	TNF-α↓IL-10↑α-SMA↓TGF-β1↓HGF↑	inhibit inflammation and renal fibrosis	[96]
Bone MSCs	LPS, CLP	Rat, HK-2	UC	/	LC3-II/LC3-I↑p-AMPK↑p62↓p-mTOR↓	enhance autophagy, anti-inflammation and apoptosis	[94]
iMSCs	IR	Mouse	UC	/	ERK 1/2 signaling↑	inhibit apoptosis, inflammation and oxidative stress	[97]
	Cisp	Mouse, HK-2	UC	/	ERK 1/2 signaling↓Bax↓Casp-3↓	anti-inflammation and apoptosis	[98]
iPSC	IR	Rat,HK-2	UC	/	SOD1↑AOX1↑SIRT1/2↑	inhibit inflammation and oxidative stress	[99]
renal MSCs	IR	Mouse	UC	CD29, CD44, CD73, α4/5/6 VEGF-A, IGF-1, integrins	VEGF-A signaling↑	promote angiogenesis	[100]

#### 2.2.1 Bone marrow MSCs (BMSCs)

BMSCs is a subset of multipotent adult stem cells known for their self-renewal, proliferation, and multilineage differentiation capabilities. BMSCs offer significant advantages over other stem cells: they are readily isolated from patients or donors, expandable *in vitro*, and possess reparative and immunomodulatory properties [48]. A host of experimental findings underscore the significant therapeutic potential of BMSCs-EVs in AKI, particularly their RNA cargo [[49], [50], [51], [52]]. Early studies using Gene Ontology Analysis revealed that in AKI mice, genes related to fatty acid metabolism in renal tissues were downregulated, while genes involved in inflammation, extracellular matrix receptors, and cell adhesion molecules were upregulated. Intravenous injection of BMSCs-EVs was able to reverse these changes, but this effect was lost in EVs derived from Drosha-Knockdown MSCs (with miRNA deficiency), suggesting that miRNAs in BMSCs-EVs have a therapeutic effect on AKI [50]. Similarly, Li et al. have shown that sEVs from BMSCs alone can mitigate inflammatory response by decreasing IL-6, TNF-α, NF-κB, and IFN-γ levels, and reduce TECs apoptosis by modulating the expressions of caspase-9, cleaved caspase-3, Bax, and Bcl-2 [51]. BMSCs-lEVs has been found to interact with TECs through CD44 and CD29 integrins, and the RNA they carry can improve renal function [52]. However, the protective effects are markedly attenuated when EVs are co-administered with RNase, as observed in the aforementioned studies. Advanced studies have shed light on the particular RNA molecules harbored by BMSCs-EVs and the intricate mechanisms by which they provide protection in AKI. Zhu et al. have revealed that sEVs enriched with miR-199a-3p can activate the AKT and ERK pathways by downregulating Sema3A, thus reducing TECs apoptosis [53]. Moreover, sEVs from BMSCs, rich in miR-1184, can suppress the inflammation in cisplatin-treated HK-2 cells by decreasing IL-1β and TNF-α expression and reverse cell growth inhibition and apoptosis by targeting FOXO4 [54]. Similarly, let-7b-5p in sEVs reduces cellular DNA damage and apoptosis by inhibiting p53 expression, with lower levels potentially increasing AKI susceptibility post-renal injury [55].

Current research is primarily centered on the functional RNA within EVs, yet occasional findings suggest that additional substances in BMSCs-EVs also contribute to the regulation of AKI. Research by Zhao et al. showed that BMSCs-EVs could elevate the levels of TFAM in damaged TECs and enhanced the stability of the TFAM-mtDNA complex, thereby reducing oxidative stress. Moreover, MSCs-EVs from TFAM knockout mice exhibited significantly lower levels of anti-inflammatory factors, indicating that MSCs-EVs mitigated mtDNA damage and inflammatory responses in injured TECs through TFAM, thus alleviating AKI [56]. A study highlighted that lEVs from BMSCs may upregulate survival genes such as Bcl-xL, Bcl2, and BIRC8, and downregulate apoptotic genes like Casp1, Casp8, and LTA in TECs, thus reducing mortality in AKI mice [57]. Besides, in IR-AKI, intravenously injection of BMSCs-sEVs can migrate directly to the kidney tissue, exhibiting anti-inflammatory properties by facilitating the polarization of M1 macrophages to the M2 phenotype, thus alleviating AKI [58]. Similarly, in rats with IR-AKI, intravenous administration of BMSCs-lEVs has been shown to inhibit TECs apoptosis and promote proliferation [59].

Intriguingly, it has been reported that EVs of different sizes, derived from BMSCs and isolated via gradient centrifugation, possess varying degrees of renal protective effects attributed to their distinct RNA profiles [60]. Thus, a deeper investigation into the precise RNA protective signals is essential. It has also been noted that when EVs from BMSCs are fractionated through gradient centrifugation into distinct groups, including sEVs and lEVs, and characterized by RNA and protein expression analyses, the molecules with regenerative potential for AKI recovery are primarily enriched in sEVs [61]. This suggests that the therapeutic impact of lEVs on AKI might be somewhat less effective than that of sEVs, owing to the differential composition of effector molecules. Moreover, the significant positive impact of BMSCs-derived EVs in other disease has also been noted. This insight not only expands our comprehension of the functionalities of BMSCs-EVs but also points to new potential research targets, suggesting their possible therapeutic value in AKI. Such as miR-149 and Let-7c have shown protective effects in myocardial IR injury by targeting FasL [62], and miR-548x-3p protects against multi-organ failure by dampening high mobility group box 1 (HMGB1), curbing inflammation, and preventing vascular endothelial cell pyroptosis in heat shock models which is easy to case AKI [63].

#### 2.2.2 Human umbilical cord MSCs (hucMSCs)

HucMSCs are multipotent stem cells isolated from neonatal umbilical cord tissue, with their secreted sEVs demonstrating significant potential in clinical applications for various diseases [64]. TECs suffering from different danger stimulations in AKI may experience diverse cell death pathways, including apoptosis, pyroptosis, programmed necrosis and autophagy, which may aggravate or alleviate renal tissue pathology. Current research indicates that hucMSCs-EVs primarily exert their protective effects by modulating various modes of cell death in TECs following AKI. Research indicates that cisplatin induces apoptosis, increases oxidative stress, and activates the p38/MAPK pathway in TECs, which can be counteracted by sEVs from hucMSCs. These sEVs also activate the ERK 1/2 pathway, reducing TECs apoptosis and oxidative stress while promoting proliferation [65]. Ju et al. have discovered that HGF mRNA within lEVs from hucMSCs can elevate HGF protein levels in TECs, promoting proliferation and reducing apoptosis in the early stages of AKI [66]. Furthermore, hucMSCs-sEVs, which accumulate on TECs via VLA-4 and LFA-1, carry miR-125 b-5p that suppresses p53 expression and modulates Bcl2 and Bax levels, reducing apoptosis [67]. MiR-148 b-3p was found in hucMSCs-EVs, which could reduce the expression of PDK4 at the transcriptional level, thus alleviating TECs injury [68]. Another miRNA presents in hucMSCs-EVs, miR-100–5p, promoted the activation of the AKT signaling pathway by targeting FKBP5, which reduced TECs apoptosis, and improved renal tissue morphology in AKI [69]. Moreover, they mitigate cisplatin's nephrotoxicity by enhancing autophagy, with rapamycin showing a comparable protective effect [70]. Further studies have discovered that the 14-3-3ζ protein in hucMSCs-sEVs interacts with ATG16L, modulating its localization in autophagosome precursors to activate autophagy and alleviate renal damage [71]. In other models, sEVs from hucMSCs carrying miR-375 have been found to target HDAC4, promoting autophagy and inhibiting T-cell apoptosis in sepsis-induced AKI, thereby exerting a protective effect [72]. Furthermore, hucMSCs-sEVs also protect TECs by inhibiting pyroptosis [73], and miR-874–3p in these EVs has been found to suppress programmed necrosis through targeting RIPK1/PGAM5 pathway in mice undergoing unilateral ureteral obstruction [74].

Beyond regulating the modes of TECs cell death, hucMSCs-sEVs have been discovered to employ a variety of mechanisms to mitigate AKI damage. High YAP protein expression promotes collagen deposition and renal fibrosis, which can be mitigated by these sEVs. They deliver CK1δ and E3 ubiquitin ligase β-TRCP to target cells, facilitating YAP ubiquitination and degradation, thus alleviating kidney fibrosis [75]. These sEVs also significantly increase miR-146 b expression in kidney tissue, which downregulates IRAK1 and inhibits the activity of pro-inflammatory signals like NF-κB [76]. Similarly, hucMSCs-sEVs have been found to suppress pro-inflammatory signals such as NF-κB and STAT3 in kidney tissue and upregulate the expression of VEGF-A and its receptors in endothelial cells, promoting angiogenesis [77]. Additionally, Hou et al. identified through proteomic analysis that hucMSCs-EVs contained SOD2, which could reduce ROS levels in renal tissues, thereby inhibiting ROS-induced mitochondrial dysfunction and subsequent cell death. Lipid nanoparticles delivering SOD2 to renal tissues could also exert protective effects against AKI [78].

#### 2.2.3 Adipose-derived MSCs (AMSCs)

AMSCs, a vital subset of MSCs, are distinguished by their multilineage differentiation potential, robust regenerative capabilities, and their ability to secrete cytokines and EVs. More accessible and plentiful than BMSCs-EVs, AMSCs-EVs have been extensively studied for the therapeutic potential in reproductive and skin regeneration disorders [79,80]. Importantly, AMSCs have also shown striking protective effects in AKI therapy. Previous research indicates that AMSCs mitigate AKI by decreasing inflammation, oxidative stress, fibrosis, and apoptosis, while promoting angiogenesis and reducing DNA damage in TECs. Recent results revealed that AMSCs-sEVs mirror these functions, and their combined application with AMSCs yields a more potent protective effect compared to AMSCs alone [81,82]. Also, AMSCs-EVs could decrease the production of mitochondrial anion superoxides in HK-2 cells under hypoxic conditions *in vitro*, reducing TECs apoptosis and ameliorating AKI *in vivo* [83]. AMSCs-sEVs have also been shown to enhance survival rates in mice by activating the sirtuin 1 (SIRT1) signaling pathway, dampening inflammatory responses and apoptosis, and improving microcirculatory disturbances [84]. Advanced studies have unveiled the specific roles of RNA molecules carried by AMSCs-sEVs in AKI, highlighting their therapeutic significance. A significant decrease in miR-342–5p levels in serum samples from AKI patients suggests its potential benefits. Further *in vivo* and *in vitro* experiments confirm that AMSCs-sEVs enriched with miR-342–5p can alleviate AKI by enhancing autophagy and suppressing inflammatory responses [85]. MiR-21–5p in EVs secreted by AMSCs could alleviate TECs injury, with miR-21–5p downregulating TLR4 expression in TECs to inhibit the NF-κB/NLRP3 signaling pathway, thus reducing inflammatory response and pyroptosis [86]. A systemic RNA analysis previously unveiled a miRNA–mRNA network between AMSCs-lEVs and damaged TECs, indicating that miRNAs in AMSCs-lEVs can reprogram genes in damaged TECs to reduce apoptosis and oxidative stress [87]. Another study reveals that the protective effect of AMSCs-sEVs on LPS-stimulated TECs is abolished by the knockdown of circVMA21, which mitigates AKI damage by targeting miR-16–5p [88]. Moreover, the therapeutic potential of allogeneic AMSCs-EVs has also been validated in a feline model of post-renal AKI. Six metabolites (carnitine, melibiose, D-glucosamine, cytidine, dihydroorotic acid, stachyose) have been identified as potential therapeutic targets of AMSCs-EVs. Regression analysis has shown correlations between these six metabolites and the levels of Scr, BUN, and blood phosphorus, suggesting they may dynamically reflect the progression of AKI [89].

#### 2.2.4 Human placental MSCs (hPMSCs)

hPMSCs are an emerging subtype of MSCs that have not been as extensively studied as other sources such as BMSCs and AMSCs. However, recent research has begun to shed light on the potential therapeutic applications of hPMSCs-derived EVs in the treatment of AKI, highlighting their significant value in this area. In recent research, EVs from hPMSCs were found to activate the Keap1-Nrf 2 signaling pathway by delivering miRNA-200a-3p to TECs, reducing mitochondria fission, stabilizing membrane potential, and increasing DNA copy number of mitochondria, thereby improving mitochondrial function [90]. Moreover, EVs produced by three-dimensional (3D)-cultured (the technique in which cells grow and interact within a three-dimensional environment) hPMSCs showed stronger anti-inflammatory and anti-apoptotic effects in AKI, with miRNA profiling revealing the most significant changes in miR-93–5p from 3D-cultured hPMSCs-EVs compared to two-dimensional (2D) culture (the technique of cultivating a monolayer of cells on a flat culture surface) [91]. Additionally, hPMSCs-EVs and EVs derived from embryonic stem cells (ESCs) have been found to promote the expression of Sox9 in renal cells after intravenous injection. Two-photon microscopy and immunohistochemical staining results showed a significant increase in the expression of Sox9 in TECs, with Sox9^+^ TECs exhibiting higher proliferative activity, thus facilitating the repair of AKI [92,93].

#### 2.2.5 Other MSCs

Beyond the aforementioned subtypes of MSCs, EVs from other types of MSCs, including bone MSCs, human umbilical cord Wharton-Jelly MSCs (hWJMSC), MSCs induced by iPSCs (iMSC), and renal MSCs, are also being explored for their potential in AKI treatment. In a sepsis-induced AKI model, bone MSCs-sEVs have shown significant protective effects, characterized by substantial amelioration of Scr and BUN levels upon intravenous administration in mice, as well as a marked reduction in kidney tissue damage [94]. Investigations reveal that following their uptake by renal cells after entering the bloodstream, bone MSCs-sEVs increase the expression of LC3-II/LC3-I and p-AMPK, while decreasing p62 and p-mTOR levels, suggesting that these sEVs mitigate inflammation and apoptosis by modulating autophagy, though the specific active components necessitate further exploration [94]. Zou X et al. discovered six miRNAs within hWJMSC-lEVs, including miR-15a, miR-15 b, miR-16, miR-195, miR-424, and miR-497, were identified as potentially protective against IR injury by correlating with the underexpression of CX3CL1 in glomerular endothelial cells, suggesting a role in reducing macrophage infiltration and inflammation, though the specific miRNA and its mechanism warrant further investigation [95]. Further research confirmed the renal protective effects of hWJMSC-lEVs in kidney transplantation from heart-beating deceased donors, where IR-induced graft failure was mitigated by these hWJMSC-lEVs, which reduced TECs apoptosis, maintained proliferation, decreased macrophage infiltration, alleviated inflammation and fibrosis in the transplanted kidney, enhancing the success rate of renal transplantation [96]. Besides, Recent study has also identified that sEVs from MSCs induced by iPSCs (iMSC) ameliorate AKI by significantly activating the ERK1/2 pathway in mice with IR-AKI [97], whereas iMSC-EVs exerted anti-inflammatory and anti-apoptotic effects by inhibiting the phosphorylation of ERK1/2 activated by cisplatin [98]. The mechanistic differences observed may arise from varying AKI models and the types of induced MSCs, yet the presence of iMSCs offers an innovative approach for the procurement and preparation of MSCs-EVs. It's worth noting that, EVs from iPSCs alone primarily improved renal injury by reducing macrophage infiltration and oxidative stress, with the similar performance than AMSCs-EVs [99]. In another research, lEVs originating from renal MSCs integrate into endothelial cells through multiple adhesion molecules, including CD29, CD44, CD73, and α4, α5, and α6 integrins. These lEVs release VEGF-A and IGF-1, which promote the proliferation of renal endothelial cells, mitigate apoptosis, and reduce vascular leakage [100].

Recent research has underscored the therapeutic impact of EVs from a spectrum of MSCs sources in AKI, ranging from prevalent sources like BMSCs and AMSCs to less explored options such as iMSC and hWJMSCs. Yet, determining the most efficacious MSCs subtype for EVs production in AKI and identifying the key substances they carry remain elusive. The mechanisms and factors dictating their efficacy are poorly charted, with only a sparse number of studies offering a cursory look into this area. There's an evident need for a more comprehensive comparative analysis to advance our understanding. Zhang et al. conducted a comparative analysis of the therapeutic efficacy between BMSCs-EVs and AMSCs-EVs in a model of LPS-induced AKI. Aligning with prior findings, both MSCs-EVs variants were effective in mitigating renal damage and enhancing kidney function. Notably, AMSCs-EVs demonstrated a more potent capacity to dampen inflammation, as evidenced by reduced transcription levels of IL-17, IL-6, TNF-α, and IFN-γ, as well as to curb oxidative stress and apoptosis [101]. In another study, Faria et al. utilized a combination of antimycin A and 2-deoxy-d-glucose to simulate ischemic injury in TECs *in vitro*. They assessed the therapeutic disparities between sEVs and lEVs sourced from BMSCs, AMSCs, and hucMSCs. The findings revealed that EVs from all three MSCs sources ameliorated the morphological integrity and mitochondrial membrane potential of injured TECs, boosted intracellular ATP synthesis, and invigorated cellular bioenergetics. However, a comprehensive evaluation identified hucMSCs-lEVs as the most efficacious, exhibiting a more pronounced impact on ATP elevation and a significant enhancement in cellular bioenergetics. This was particularly evident in the increased levels of glycolytic intermediates, NADH, and NADPH, alongside a decrease in oxidative metabolites such as NAD^+^ and NADP^+^ [102].

On the foundation of current research conclusions, further exploration is essential. Firstly, the causes of AKI are varied and involve distinct pathological mechanisms. Consequently, EVs from the same subtype of MSCs may play different therapeutic roles in AKI triggered by different etiologies. Similarly, EVs from different MSCs sources may also show significant differences in therapeutic efficacy in AKI caused by the same cause. This suggests that the findings from a single experiment cannot comprehensively represent the overall therapeutic effectiveness of EVs from a specific MSCs subtype in AKI treatment. Secondly, the outcomes of animal studies and *in vitro* cell experiments do not directly mirror the therapeutic effects of MSCs-EVs in humans, necessitating further validation in human studies. Lastly, various factors, including EVs extraction techniques, concentration used, and operational errors, can influence experimental results, adding to the complexity of the findings. Therefore, a comparative analysis of EVs secreted by different MSCs subtypes in AKI treatment is not only significant but also demands deeper investigation. Moreover, there may be distinct therapeutic effects between sEVs and lEVs from the same cellular origin, indicating the need to consider the therapeutic differences of EVs of varying sizes, which further broadens and deepens the scope of research. Concurrently, a cost-benefit analysis of acquiring EVs from different sources is a critical aspect for future research, and all these issues merit further study and exploration.

### 2.3 Stem/progenitor cells-derived EVs in AKI treatment

In addition to MSCs-EVs, those from other stem/progenitor cells have also demonstrated significant renoprotective effects. A meta-analysis indicated that EVs originating from stem/progenitor cells could ameliorate renal function following AKI through multiple mechanisms, including the reduction of apoptosis, promotion of cell proliferation, mitigation of inflammatory damage and renal fibrosis, enhancement of angiogenesis, and inhibition of oxidative stress [103] (Fig. 3, Table 3).

**Table 3:** The role of stem/progenitor-derived EVs for AKI therapy.

Source	Condition	Model	Isolation	Cargo	Mechanism	Outcome	Ref
EPCs	IR	Rat	UC	miR-126, miR-296	/	promote angiogenesis	[106]
	CLP	Mouse	Isolation kit	miR-126	HMGB1↓VCAM1↓	suppress renal vascular leakage	[109]
	CLP	Rat	Isolation kit	miR-21–5p	RUNX1↓	inhibit inflammation, apoptosis and oxidative stress	[110]
	CLP	Mouse, HK-2	UC	miR-93–5p	KDM6B↓H3K27me3↑TNF-α↓	suppress vascular leakage, inflammation and apoptosis	[111]
ECFCs	IR	Mouse	UC	miR-486–5p	PTEN↓p-AKT↑TNF-α↓	suppress vascular leakage, inflammation and apoptosis	[114,115]
hAECs	IR	Mouse, HK-2	UC	functional proteins	/	anti-apoptosis, M2 polarization and angiogenesis	[116]
	Cisp	Mouse, HK-2	UC	/	TNF-α/MAPK↓ Caspase signaling↓	anti-inflammation and apoptosis	[117]
	CLP	Mouse, HK-2	UC	/	NF-κB pathway↓ VCAM-1↓	anti-inflammation, stabilize endothelium	[118]
hUSCs	IR	Rat, HK-2	Isolation kit	miR-146a-5p	IRAK1↓NF-κB↓	anti-inflammation	[122]
	IR	Rat, HK-2	UC	miR-216a-5p	PTEN↓p-AKT↑	anti-apoptosis	[123]
	IR	Mouse, HK-2	Isolation kit	lncRNA TUG1	TUG1-SRSF1 interaction ACSL4↑	*anti*-ferroptosis	[124]
	IR	Rat, HK-2	UC	circ DENND4C	miR-138–5p↓FOXO3a↑	*anti*-pyroptosis	[126]
ESCs	IR	Mouse, HK-2	UC	/	activate Sox9^+^ cells	promote angiogenesis and proliferation	[93]
SCAPs	Cisp	NRK-52 E	UC	/	NF-κβ↓IL-1β↓p53↓Bcl2↑Bax↓	anti-inflammation and apoptosis	[128]

#### 2.3.1 Endothelial progenitor cells (EPCs)

EPCs have been widely studied for their potential in treating various diseases including vascular diseases, ischemic and inflammatory conditions [104], and previous studies have indicated that EPCs-EVs could activate quiescent endothelial cells [105] and inhibit apoptosis, thus protecting the kidney from IR injury [106,107]. These effects were primarily attributed to the RNA signals delivered by EVs. The RNA cargo within these lEVs helps mitigate AKI by promoting TECs proliferation, reducing apoptosis, decreasing inflammatory cell infiltration, and encouraging angiogenesis. The ameliorative impact of EPCs-lEVs on AKI is markedly reduced upon nonspecific RNA signal ablation within the lEVs or targeted disruption of angiogenesis-associated miRNAs, such as miR-126 and miR-296 [106,108]. In the context of sepsis, a condition that can precipitate acute multi-organ failure, EPCs-sEVs are enriched with miR-126–5p and 3p. These microRNAs specifically target HMGB1 and vascular cell adhesion molecule 1 (VCAM-1) in microvascular endothelial cells, preserving the integrity of the capillary endothelium and mitigating vascular leakage in organs such as the kidney [109]. Furthermore, miR-21–5p within these sEVs has been identified to downregulate RUNX1, which ameliorates AKI in rats, although the exact mechanisms of RUNX1 regulation by miR-21–5p are not yet fully understood [110]. MiR-93–5p which exist in hPMSCs-EVs [91] was also enriched in EVs from EPCs, which could silence KDM68, activate H3K27me3, inhibit the TNF-α signaling, and alleviate kidney injury [111]. These suggest that hPMSCs and EPCs may protect the kidney from acute injury through similar mechanisms. Moreover, EPCs-EVs integrated with renal glomerular endothelial cells and podocytes through L-selectin. Within glomerular endothelial cells, they maintained the stability of the capillary structure by inducing the release of growth factors such as VEGF-A and HGF. Under inflammatory stimuli, these EVs reduced the apoptosis of glomerular endothelial cells by inhibiting oxidative stress and decreased the expression of adhesion factors like ICAM-1, VCAM-1, and *E*-selectin, thereby reducing the adhesion of pro-inflammatory cells. In podocytes, EPCs-EVs also reduced damage caused by pro-inflammatory factors and the complement system, which were abolished by RNase, demonstrating that RNA within EVs has an impact on maintaining the integrity and stability of the glomerular filtration barrier, preventing damage from pro-inflammatory factors and the complement system [112]. In addition, human cord blood endothelial colony-forming cells (ECFCs), as primitive endothelial progenitors, are being explored for their capacity to alleviate AKI by inhibiting endothelial cell apoptosis [113]. Advanced studies suggest that ECFCs-sEVs may exert their therapeutic effect in AKI by harboring miR-486–5p, which can inhibit PTEN and activate the AKT pathway, thereby reducing apoptosis in endothelial cells and TECs [114,115]. Additionally, miR-486–5p has shown to diminish the expression of TECs injury markers and TNF-α signaling, modulating the expression of metabolic genes in glomerular endothelial cells [115].

#### 2.3.2 Human amniotic epithelial cells (hAECs)

sEVs from alternative stem cells, including hAECs, have shown considerable therapeutic potential in AKI. Obtained from the amniotic membrane postnatally, hAECs are readily accessible and possess low immunogenicity and high tissue compatibility, endowing them with immune-privileged properties. Studies have demonstrated that sEVs derived from hAECs can shield mouse kidneys from AKI triggered by diverse factors. In IR AKI models, these sEVs have been shown to diminish apoptosis in TECs and facilitate the polarization of macrophages towards an M2 phenotype. Proteomic analyses have uncovered that hAECs-sEVs are enriched with proteins that participate in extracellular matrix organization, growth factor signaling, cytokine production, and immune modulation [116]. Cisplatin, a broadly utilized chemotherapeutic agent for oncology, but has nephrotoxic side effects. Surprisingly, sEVs from hAECs have been found to markedly alleviate cisplatin-induced renal damage. This is largely attributed to their ability to suppress the TNF-α/MAPK and caspase signaling pathways, thereby reducing renal inflammation and TECs apoptosis without promoting tumor growth or compromising the antineoplastic efficacy of cisplatin [117]. In sepsis-induced AKI, sEVs secreted by hAECs have dual benefits: they enhance endothelial cell adhesion, curb excessive endothelial cell activation, and preserve the integrity of renal capillaries. Concurrently, they inhibit the NF-κB signaling pathway, thereby mitigating inflammatory responses [118].

#### 2.3.3 Human urine-derived stem cells (hUSCs)

hUSCs exhibit multipotency, proliferation, and immune-modulatory capabilities akin to MSCs, with the added advantage of easy retrievability from urine. The EVs, secreted by hUSCs have been extensively investigated for therapeutic potential across a spectrum of diseases, demonstrating significant promise for clinical utility [119]. Preliminary studies have highlighted the protective effects of hUSCs in renal pathologies such as AKI and CKD. These effects are evidenced by improved Scr and BUN levels, reduced inflammatory cytokines, and increased anti-inflammatory mediators within the renal tissue microenvironment, as well as the inhibition of apoptosis in TECs [120,121]. Nevertheless, the precise mechanisms remain to be fully elucidated. Advanced research has identified miR-146a-5p within hUSCs-sEVs, which can target and suppress AKI, impede the NF-κB signaling cascade, reduce inflammatory responses and cell infiltration, thereby safeguarding TECs from injury [122]. Additionally, miR-216a-5p, abundant in hUSCs-sEVs, reduces PTEN levels and activates the AKT pathway, leading to decreased TECs apoptosis [123]. Ferroptosis and pyroptosis within renal tissue are also modulated by hUSCs-sEVs. The lncRNA TUG1, highly expressed in these sEVs, interacts with the RNA-binding protein SRSF1 to stabilize ACSL4 mRNA, thereby inhibiting ferroptosis in TECs and protecting renal tissue [124]. In another study, lncRNA TUG1 was shown to upregulate *E*-cadherin levels by targeting miR-494–3p, reducing TECs apoptosis [125]. Furthermore, Circ DENND4C, a circRNA with significantly reduced expression in AKI rat kidney tissue, was found to be upregulated by hUSCs-sEVs, restoring its levels and mitigating AKI by inhibiting pyroptosis through the miR-138–5p/FOXO3a signaling axis [126].

In summary, within the realm of AKI-related EVs research, stem/progenitor cells emerge as pivotal sources of therapeutic EVs. These cells generate EVs that positively influence AKI through a spectrum of mechanisms, including the reduction of apoptosis in endothelial and TECs, mitigation of inflammation, and attenuation of fibrosis. Notably, hUSCs, which are adult stem cells with stem cell-like properties, are isolated and cultured from human urine. They offer the benefits of ease of access and non-invasiveness, demonstrating substantial potential in the research on AKI treatments. Furthermore, EVs derived from endothelium-related stem/progenitor cells possess distinct advantages in preserving the integrity of renal vascular endothelial cells and curbing vascular leakage. This suggests that EVs from various origins have their own therapeutic strengths in AKI treatment. However, current studies have also uncovered some commonalities, such as the presence of miR-21 in EVs from TECs, AMSCs, and EPCs [26,86,110]. These EVs, whether acting through paracrine or systemic circulation, can elevate miR-21 levels in TECs, thus influencing the outcomes of AKI. Consequently, assessing the potential of EVs from different sources in AKI treatment essentially involves uncovering the capabilities of specific effector molecules. Given that different effector molecules may exhibit preferences in their signaling pathways and biological effects, pinpointing the specific functional molecules and exploring the potential for their combined application could significantly enhance clinical translation. Additionally, further investigation into the role of EVs from other stem/progenitor cell sources in AKI is equally vital. Renal interstitial fibrosis is a key pathological change in the transition from AKI to CKD, and EVs from human liver stem cells (HLSCs) could suppress the expression of profibrotic genes and epithelial-mesenchymal transition (EMT), thereby halting the progression from AKI to CKD [127]. Recent investigations have also highlighted the role of CD63^+^ and CD81^+^ sEVs from stem cells of the apical papilla (SCAPs). These sEVs, by preconditioning the NRK-52 E cell line, enhance resistance to cisplatin-induced toxicity through the suppression of inflammatory responses and apoptosis [128].

### 2.4 Other sources derived EVs in AKI progression and treatment

Aside from the EVs mentioned above, other cells derived EVs are also involved in the pathophysiological processes of AKI, and investigating these EVs may offer novel therapeutic strategies (Table 4). Macrophages are key immunological players in inflammation and are crucial in the tissue damage and repair processes associated with numerous diseases. Recent research highlights the complex connection between macrophages and renal diseases [[129], [130], [131]], emphasizing their critical role in kidney pathologies and the importance of macrophage-derived sEVs in AKI. Previous studies have demonstrated that injured TECs can activate macrophages via sEVs [27,28,30], and conversely, activated macrophages can exacerbate TECs damage through the communication of sEVs. Numerous studies have highlighted the significant regulatory role of miR-155, carried by macrophage-derived EVs, in the exacerbation of AKI. Results showed that activated macrophages release sEVs containing miR-155, which are taken up by TECs, directly target SOCS1, and enhance NF-κB signaling, thereby intensifying inflammation and AKI progression [132]. Consistent with this, another research has proved that upregulation of miR-155 in TECs also promotes apoptosis and inhibits proliferation by suppressing TCF4/Wnt/β-catenin signaling, worsening renal damage [133]. Furthermore, miR-155 can negatively regulate the expression of TRF1 and CDK12 in TECs, increasing telomere dysfunction and DNA damage [134]. Thus, downregulating miR-155 expression or blocking sEVs-derived miR-155 may represent a viable therapeutic strategy for AKI. Except for miR-155, another miRNA miR-195a-5p in activated macrophage sEVs, can induce mitochondrial dysfunction in TECs, aggravating cell injury and AKI [135]. In macrophages with impaired autophagy, sEVs contain high levels of miR-195a-5p, which targets SIRT3 in TECs. These EVs harms mitochondria and exacerbates renal injury. Inhibition of miR-195a-5p or overexpression of SIRT3 can mitigate TECs injury [136]. Moreover, activated macrophages infiltrate the glomeruli and secrete sEVs that harm glomerular endothelial cells during AKI. Acid sphingomyelinase (ASM) inhibitors or ASM gene knockout can reduce the production of macrophage sEVs to mitigate kidney injury [137]. These findings suggest that activated macrophages can inflict damage on TECs and endothelial cells via sEVs, hastening the development of AKI. However, considering the dual nature of macrophages with both pro- and anti-inflammatory subtypes, the anti-inflammatory M2 subtype predominantly exerts a protective effect in AKI [138]. Compared to M1 macrophages, M2 macrophage-derived sEVs are enriched with miR-93–5p, which can modulate TXNIP signaling in TECs, inhibit pyroptosis, and alleviate AKI [139]. Yet, studies on the precise functions of EVs secreted by distinct macrophage subtypes in renal injury and repair are notably lacking. This research underscores the need to explore the role of EVs from different macrophage subtypes in greater depth.

**Table 4:** The role of other cell-derived EVs in AKI progression.

Source	Condition	Model	Isolation	Cargo	Mechanism	Outcome	Ref
Mø	IR	Mouse, mTECs	UC	miRNA-155	SOCS-1↓NF-κB↑	promote inflammation	[132]
	Cisp	Mouse, mTECs	UC	miR-195a-5p	/	mitochondrial dysfunction	[135]
	Cisp	Mouse	UC	miR-195a-5p	SIRT3↓	inhibit autophagy	[136]
	LPS	Mouse	UC	/	VCAM-1↑NLRP3↑	endothelial injury	[137]
	CLP	Mouse	Isolation kit	miR‐93–5p	TXNIP↓NLRP3↓	*anti*-pyroptosis	[139]
Platelets	CLP	Mouse, HK-2, mTECs	UC	ARF6	p-ERK↑p-Smad3↑p-p53↑	promote apoptosis, inflammation and oxidative stress	[140]
	LPS	Mouse	Isolation kit	miR-223–3p	NLRP3↓	*anti*-pyroptosis	[141]
C2C12	CLP, LPS	Mouse, mTECs	UC	miR-21	PDCD4↓NF-κB↓PTEN↓AKT↑	anti-inflammation and apoptosis	[144]
Circulating EVs	Cisp	HK-2	UC	miR-500a-3P	p-MLKL↓	reduce necroptosis	[145]
FRCs	CLP	Mouse	UC	CD5L	PINK-Parkin pathway↑NLRP3↓	promote mitophagy, *anti*-pyroptosis	[150]

In the context of sepsis, elevated platelet counts are indicative of infection severity. Studies reveal that platelet-derived EVs boost ARF6 in TECs, initiating the ERK/Smad3/p53 pathway and intensifying apoptosis, inflammation, and oxidative stress, which exacerbates AKI [140]. In tandem, studies reveal a decline in miR-223–3p within platelet-sEVs, which curbs endothelial pyroptosis by antagonizing NLRP3-driven inflammasomes. Decreased miR-223–3p expression, in turn, fuels inflammation in AKI [141].

An emerging body of evidence underscores the significant inter-organ interactions in AKI's initiation and progression, with further exploration needed to unravel the specific mechanisms. EVs, serving as conduits for cellular and tissue communication, are increasingly recognized for their essential influence in this process. Trauma, particularly thoracic injury, prompted the local damaged tissue to produce many cytokines. These cytokines stimulated endothelial cells to generate a plethora of EVs containing pro-inflammatory substances, which entered the blood stream. These EVs carried a wealth of transcriptional information, including ICAM-1, VCAM-1, *E*-selectin and cytokines, affecting the vascular endothelium throughout the body. They promoted interactions between the endothelium and inflammatory cells, disrupted vascular barriers, and may have led to damage in distant organs, such as AKI. Therefore, blocking endothelial cells-derived EVs or their carried transcriptional signals in the circulation of patients with trauma could potentially prevent the occurrence of systemic multi-organ failure [142]. Clinically, an increase in hemoglobin-rich erythrocyte-derived sEVs was observed in the plasma of 30 patients post-cardiopulmonary bypass (akin to IR injury). Intravenous injection of these sEVs into rats induced AKI approximately 72 h later, with persistent renal tubular damage and interstitial fibrosis observed up to 21 days post-injection [143]. A study found that preconditioning mice with femoral artery ischemia significantly improves sepsis-associated AKI. Further investigation indicates that sEVs derived from ischemia-preconditioned myocytes (C2C12 cells), enriched with miR-21, are circulated to TECs and target the PDCD4/NF-κB and PTEN/AKT pathways, thereby exerting anti-inflammatory and anti-apoptotic effects [144]. Additionally, circulating EVs of unknown origin can also influence the course of AKI. In AKI patients, circulating levels of hsa-miR-500a-3P are significantly decreased. *In vitro*, hsa-miR-500a-3P targets MLKL signaling in HK-2 cells to mitigate necroptosis and inflammatory responses following IR injury [145]. Similarly, liposome-encapsulated hsa-miR-500a-3P alleviates acute injury in HK-2 cells induced by cisplatin [146]. Circulating sEVs in septic mice induce apoptosis and pyroptosis in TECs, triggering AKI, but renal tissue-expressed Surfactant protein A (SP-A) can ameliorate the damage caused by these sEVs [147]. In conclusion, sEVs in AKI patients' circulation serve both pathogenic and protective roles, with origins that are likely complex. Elucidating these mechanisms will be instrumental in advancing AKI treatment strategies.

The therapeutic potential of EVs derived from specific tissues in the treatment of AKI is increasingly capturing attention. Neonatal tissues exhibit remarkable regenerative capacity. Consequently, scientists have designed neonatal-tissue-derived EVs therapy (NEXT), which has demonstrated potent tissue repair capabilities in AKI models [148]. Adiponectin (APN), a circulating protein, facilitates the production of sEVs by perivascular cells in a T-cadherin-dependent manner, which is crucial for maintaining renal capillary stability, and deficiency in APN or T-cadherin leads to increased renal vascular permeability and tubular injury [149]. Fibroblastic reticular cells (FRCs), specialized cells in lymphoid organs, release sEVs containing CD5L that promote PINK1-dependent mitophagy in damaged TECs, suppress pyroptosis, limit inflammasome formation, and therefore reduce inflammation [150].

## 3 EVs as potential diagnostic biomarkers in AKI

Currently, the clinical diagnosis of AKI is predominantly based on levels of Scr and BUN. The identification of biomarkers with enhanced specificity and sensitivity is becoming increasingly vital for AKI detection [7]. AKI sparks significant renal changes that ripple through the body, affecting distant organs and tissues, with a notable impact in sepsis-associated AKI where systemic effects are particularly pronounced. These interactions are mirrored in the circulatory system through changes in EVs and their cargo. By closely monitoring these EVs fluctuations, we can detect AKI at its earliest stages, facilitating prompt medical intervention. Silva et al. reported that sEVs-containing miR-181a-5p and miR-23 b-3p in serum are significantly elevated in AKI, which is associated with transcription factors that modulate pro-inflammatory cytokine expression. Given LPS's link to inflammatory responses, the early changes in these miRNAs could be instrumental in identifying sepsis-related AKI [151], though their specificity warrants further investigation. Elevated levels of *E*-selectin^+^ lEVs in the circulation of patients with AKI has also been found [152]. Furthermore, research indicates that platelet-derived EVs (P-EVs) have the potential to serve as early warning biomarkers for AKI, aiding in guiding patient treatment and prognosis. Patients with urosepsis showed significant increases in serum inflammatory cytokine levels which was correlated with increased P-EVs, suggesting that P-EVs may be involved in the pathogenesis of AKI by mediating the release of inflammatory cytokines [153]. Also, there is an inverse relationship between the levels of platelet-derived CD42a^+^ lEVs and the severity of renal impairment [154].

Urinary EVs and their cargo can be non-invasively harvested, offering potential as novel bioindicators for predicting and evaluating renal pathology. Nucleic acids, a key component of EVs, have been established as promising biomarkers for kidney disease [155]. Further research has uncovered additional nucleic acid constituents within urinary EVs that can signal AKI pathogenesis. For example, urinary EVs-derived miRNA-29c correlates with the extent of renal fibrosis, potentially serving as an early biomarker for the progression from AKI to CKD [156]. In IR-AKI, miRNAs in urinary sEVs exhibit stage-specific alterations. During the acute injury phase, miR-16, miR-24, and miR-200c are notably elevated, while in the early recovery phase, miR-9a, miR-141, miR-200a, miR-200c, and miR-429 are significantly upregulated. These miRNAs converge on the common target mRNA Zeb 1/2, which is implicated in TGF-β-driven renal fibrosis [157]. In patients with scrub typhus-associated AKI, miRNA-21 levels in urinary sEVs are markedly increased and inversely correlate with white blood cell counts and estimated glomerular filtration rate (eGFR), indicating that miRNA-21 in urinary sEVs could be a specific diagnostic biomarker for scrub typhus-related AKI [158].

Urinary EVs encompass a spectrum of molecules beyond nucleic acids, including metabolites and transcription factors, which also demonstrate potential as AKI biomarkers. Recent metabolomic analyses have identified at least 396 metabolites in urine EVs from rats with AKI induced by cisplatin and aminoglycosides, pinpointing 65 metabolites that could serve as biomarkers reflecting cellular damage within the kidney [159]. Proteomic studies have similarly identified 251 proteins in urinary EVs from patients with vancomycin-induced AKI, highlighting the need for further research to elucidate their roles in renal injury [160]. Early studies have shown that elevated WT-1 levels in urinary EVs mirror early podocyte damage, with ATF3 levels significantly increased, reaching 60-fold that of normal controls. Notably, changes in urinary EVs' ATF3 and NGAL precede alterations in Scr and BUN, underscoring their value as early diagnostic biomarkers for AKI [34,161,162]. Furthermore, urine NHE3 has been recognized as a valuable diagnostic marker for AKI [163]. Subsequent research has indicated that NHE3 levels in urinary sEVs are markedly elevated in cisplatin-, IR-, diuretic-, angiotensin receptor blocker-, and sepsis-associated AKI, exhibiting greater sensitivity than Scr changes [164]. Fetuin-A [165] and AQP1/2 [166] in urinary sEVs have also emerged as potential AKI biomarkers. In both rat IR models and cisplatin-induced AKI patients, Fetuin-A in urinary EVs was notably increased. In AKI, AQP1 levels in urinary EVs slightly rose within 24 h, while AQP2 levels plummeted, suggesting that combined monitoring of AQP2 and AQP1 changes can realize diagnosis of cisplatin-induced renal damage, preceding traditional hematological markers.

## 4 Advances in EVs isolation for AKI

While our earlier discussions have thoroughly explored the potential of EVs as candidates for the treatment and diagnosis of AKI, translating these insights into clinical practice requires overcoming the challenge of producing EVs that possess five critical attributes: high purity, rapid preparation, scalability, cost-effectiveness, and safety. These traits are essential for the effective clinical utilization of EVs. It is well known that EVs are ubiquitous in human body fluids, particularly in blood and urine [167], offering a practical avenue for their use in AKI diagnostics. They hold a significant advantage over invasive biopsy procedures due to their easier accessibility [168]. In the realm of AKI therapy, our previous conversations have highlighted the substantial potential of EVs derived from MSCs and other stem/progenitor cells, which are typically sourced from cell or tissue culture supernatants [169]. Consequently, the isolation of EVs from relevant body fluids or culture media that meet the aforementioned criteria to facilitate clinical translation of AKI is a research question that merits further exploration [170].

Technological strides have revolutionized the field of EVs separation techniques. Kumar et al. have meticulously categorized these methods based on their underlying principles [171]. The prevailing conventional techniques can be broadly classified into four categories: a. Size/density-based centrifugation methods, such as ultracentrifugation (UC) [172], size exclusion chromatography (SEC) [173], and density gradient ultracentrifugation [174]; b. Filtration-based separations, including ultrafiltration (UF) [175] and Exodisc [176]; c. Precipitation-based methods, like polymer precipitation [177], and commercial kits like urine exosome RNA isolation kit and total exosome isolation solution [178]; d. Affinity-based separations, featuring immunocapture [179] and magnetic beads [180]. Each method has its merits, but also significant limitations. For instance, centrifugation can compromise EV integrity and bioactivity, with low yields and high equipment costs [172,[181], [182], [183]]. Precipitation methods, while simple, result in low purity and risk of polymer and protein contamination [172,177,184]. Ultrafiltration, despite its large-scale and rapid separation capabilities, faces issues of long processing times, low purity and recovery rates, and protein contamination [172,[185], [186], [187], [188]]. Affinity separations are constrained by factors like bead binding capacity, antigen exposure, and epitope stability [179,189]. Thus, optimizing existing methods is imperative. Improvements in EVs separation are focused on two fronts: innovating existing techniques and integrating new technologies to enhance their performance. Tangential flow filtration (TFF), for example, offers significant advantages over direct flow filtration (DFF) with its cross-flow design that minimizes particle accumulation on membranes, ensuring efficient separation and vesicle integrity while being scalable [186,190]. Notably, Aopia Biosciences' NanoEX technology has gained attention for its asymmetric nano-porous membranes, which prevent clogging and protein contamination, outperforming TFF in yield, purity, and convenience. Secondly, there is an ongoing exploration of entirely new separation techniques. Professor Mengsu Yang's team has highlighted cutting-edge methods with high application potential [191]. These include: a. Microfluidic techniques [184], which precisely control particle behavior in fluids and have been combined with nanomaterials [192] and magnetic beads [193] to create platforms that automate EVs extraction from small samples with high purity, albeit at a higher equipment cost. b. Nanotechnology [194], leveraging the large surface area of nanomaterials to provide numerous binding sites for EVs, enhancing capture efficiency. Studies have reported the combination of nanomaterials with microfluidics for EV separation in various cancer diagnostics [[195], [196], [197]]. c. Dielectrophoresis (DEP) technology, which uses electric fields to separate particles based on their dielectric properties [198], showing potential in tumor diagnostics when combined with other techniques [[199], [200], [201]]. However, electric fields may affect EV functionality, making DEP more suitable for diagnostics than therapy. d. Beyond these, other technologies such as acoustopfluidics [202], asymmetrical flow field-flow fractionation [203], and anion exchange chromatography [204] also demonstrate significant potential in EVs separation. Here, we focus on core technologies relevant to AKI diagnosis and treatment, without delving into every other technique.

Research on EVs in AKI has seen thrilling advancements, yet the reproducibility and consistency of findings across studies are suboptimal, largely due to the variety of EVs isolation methods used. The choice of separation technique is foundational to EVs research, dictating the characteristics of the material for subsequent analysis [177]. In AKI diagnostics, blood and urine are ideal samples for “liquid biopsy," with urinary EVs reflecting the health and disease progression of the urinary system, particularly the kidneys [205]. However, isolating EVs from these fluids is fraught with challenges: blood contains a multitude of components such as cells, free nucleic acids, and lipoproteins. Components like high-density lipoprotein (HDL) and platelets, similar in size and density to EVs, complicate their isolation [[206], [207], [208], [209]]. Similarly, urinary inorganic particles, proteins, and bacteria, especially in urinary disorders, accumulate in large amounts, increasing the difficulty of EVs isolation [210,211]. In AKI treatment research, cell culture supernatants are the most common source of EVs, which include MSCs and stem/progenitor cells. These cells not only produce EVs but also secrete other soluble proteins and cytokines during culture, adding to the complexity of EVs separation [167]. Dong et al.'s study compared three mainstream EVs isolation techniques—UC, precipitation, and SEC + UF, along with the emerging microfluidic tangential flow filtration device—Exodisc, for their ability to extract EVs from cell culture supernatants, human plasma, and urine, focusing on yield and purity as key indicators [212]. In terms of EV yield, precipitation methods were the most productive in culture supernatants, followed by Exodisc. UC and SEC + UF yields were similar, approximately one-fourth that of precipitation methods. In urine, Exodisc had the highest yield, followed by SEC + UF, with UC yielding the lowest, only one-tenth to one-twentieth of Exodisc. In plasma, there was a significant difference in yields among methods, with precipitation and Exodisc yields being similar and about 100 times higher than UC. The study also found that the purity of EVs extraction from different samples varied due to differences in content and extraction methods. Overall, urinary EVs had the highest purity, followed by culture supernatants and plasma. Specifically: a. UC had the highest purity in culture supernatants and plasma but lower purity in urine due to easy co-precipitation with Tamm-Horsfall protein (THP)/THP-EVs complexes. b. SEC + UF showed balanced performance across samples, especially high purity in culture supernatants and urine, but in plasma, although the yield was higher than UC, it contained many non-EV particles. c. Precipitation methods, due to the precipitation of all soluble particles [[213], [214], [215]], had the lowest purity. d. Exodisc, as a new technology, combines the efficiency of TFF with the portability and user-friendliness of microfluidic devices [176,216], had average purity in culture supernatants, high purity in urine, and low purity in plasma. Despite this, Exodisc performed best in EVs yield and operated quickly, suitable for EVs separation in various downstream experiments, especially showing clinical application potential in urine samples [212]. Notably, beyond the classic methods, the potential role of some emerging EVs isolation technologies in AKI diagnosis and treatment is becoming increasingly apparent. The EXODUS technology developed by Liu et al. at Wenzhou Medical University integrates negative pressure conversion technology with nano membranes and high-low frequency vibrations on devices, effectively reducing separation time while significantly improving EVs yield and purity. Moreover, EXODUS has shown good compatibility, efficiently isolating EVs from various biological samples such as plasma, cell culture fluid, and urine. In clinical sample processing, the technology extracted EVs from the urine of 113 patients with urinary system diseases and analyzed their long RNA profiles, proving the efficiency and consistency of EXODUS in handling a large number of clinical samples [217]. Overall, when applying EVs in AKI diagnosis and treatment, researchers need to carefully select the most appropriate EVs isolation technology based on sample characteristics, subsequent analysis requirements, and actual working conditions. Future research should focus on exploring new methods for efficiently extracting EVs from plasma, culture medium, and urine, with the aim of developing more optimized extraction strategies. At the same time, considering that most current blood and urine samples are taken from healthy donors, future research should also pay more attention to EVs extraction technologies under disease conditions to obtain results with greater clinical application value.

## 5 Engineered EVs for enhanced AKI therapy efficiency

EVs, as the workhorses of cell-to-cell communication at the nanoscale, have become a focal point of research and engineering due to their exceptional biocompatibility, lack of toxicity, minimal immunogenicity, and their ability to reduce the side effects of drugs, positioning them as a versatile tool for treating a myriad of diseases [218,219]. However, when it comes to their direct therapeutic impact, natural EVs encounter several hurdles, such as limited natural production and suboptimal purity. Moreover, the *in vivo* application of EVs is hampered by a fleeting circulation half-life, inadequate targeting capabilities, and a lack of payload specificity, all of which markedly diminish the therapeutic potency of natural EVs [[220], [221], [222], [223], [224], [225], [226]]. In this section, we shift our focus to the advancements in engineered EVs and their enhanced potential in the treatment of AKI. Given that the purification and isolation of EVs have been thoroughly discussed earlier, we will not rehash those details but instead distill our summary into four key areas: (1) Production and yield enhancement, (2) Circulation and stability optimization, (3) Targeting and delivery precision, and (4) Therapeutic potency augmentation.

### 5.1 Production and yield enhancement

**Fig. 4:**
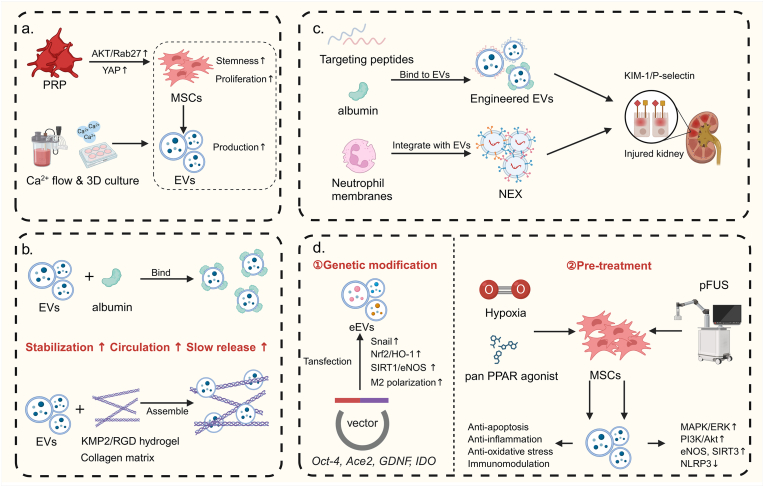
Four engineering strategies to augment the efficacy of EVs in AKI therapy. a. Enhance the production efficiency of EVs by using PRP, which increases the stemness and proliferation of MSCs, thereby promoting EVs secretion; 3D culture systems and calcium ion currents can significantly boost EVs yields from MSCs. b. Extend the *in vivo* half-life of EVs by complexing them with KMP2, RGD hydrogels, or collagen matrices, which protect EVs from degradation and facilitate a sustained release at the site of injury. c. Improve tissue specificity of EVs by incorporating attachment peptides, which target injured kidneys expressing KIM-1, P-selectin and other molecules can be utilized for enhanced targeting. NEX, a complex of EVs and neutrophil membranes, leverages neutrophil membrane proteins for targeted delivery to injured renal tissues. d. Enhancing therapeutic potency can be achieved through two strategies: Genetic modification, which involves overexpressing *Oct-4*, *Ace2*, *GDNF*, and *IDO* in MSCs to boost the therapeutic impact of EVs via multiple mechanisms; and Pre-treatment, which improves EVs secretion by hypoxia or pan PPAR agonists, thereby reducing apoptosis, inflammation, oxidative stress, and modulating immune responses. Additionally, administering pFUS to MSCs before EVs treatment optimizes outcomes by activating the MAPK/ERK, PI3K/Akt, and eNOS/SIRT3 pathways, inhibiting inflammasome activation.

Securing a robust supply of EVs is essential for their therapeutic deployment in AKI. While cutting-edge separation technologies have notably improved EVs yield and purity [217,227], strategies to enhance EVs production at the cellular level are also under investigation. A 3D culture system utilizing a hollow fiber bioreactor has been engineered to boost EVs output without compromising the integrity of MSCs-EVs, achieving a 15.5-fold increase in yield compared to conventional 2D culture techniques [91,228]. Moreover, a calcium ion-flow based bioreactor has demonstrated the capacity to significantly amplify MSCs-EVs production, reaching yields sevenfold higher than those obtained under static culture conditions [229]. It has been discovered that platelet-rich plasma (PRP) not only bolsters the regenerative potential of MSCs by upregulating YAP expression but also stimulates the secretion of EVs via the AKT/Rab27 pathway, positively impacting renal function and histopathological features in glycerol-induced AKI rat models [230]. Histologically driven research has introduced a therapeutic strategy leveraging EVs sourced from neonatal tissues. This method offers a streamlined and efficient protocol for isolating high-yield, high-purity EVs, and the EVs capitalize on their tissue-specific reparative capabilities to address renal damage stemming from AKI [148]. By amalgamating these cultivation conditions or stimulatory factors, the production of EVs can be further augmented, expanding the therapeutic horizons for AKI treatment (Fig. 4a).

### 5.2 Circulation and stability optimization

To fully realize the therapeutic potential of EVs in treating AKI, it is essential to enhance their stability in the circulatory system. With a circulatory half-life of only about 4 h, EVs' brief presence limits their sustained action and therapeutic effectiveness [231,232]. Therefore, improving EV stability and extending their half-life in circulation are critical challenges for maximizing their therapeutic efficacy. Researchers have investigated multiple strategies to bolster EVs stability [233,234]. In AKI therapy, albumin emerges as a superior protein carrier, extending the residency time of EVs within the body. Evidence suggests that albumin-conjugated EVs exhibit an extended half-life [235], and albumin's renal tissue affinity facilitates uptake by TECs [236], thereby enhancing the therapeutic potential of albumin-bound EVs in cisplatin-induced AKI mouse models [237]. Inspired by sustained-release drug delivery systems, scientists have engineered a matrix metalloproteinase-2 (MMP2)-responsive hydrogel system using the KMP2 peptide (Ac-KLDLPVGLIGKLDL-CONH2) to shield MSCs-EVs from degradation. This system preserves EVs bioactivity and sustains their release over 72 h, effectively mitigating IR induced renal damage [238]. Additionally, a hydrogel strategy incorporating the RGD (Arg-Gly-Asp) peptide, known for its integrin-binding properties, protects the let-7a-5p miRNA within MSCs-EVs from degradation, playing a pivotal role in renal recovery [239]. Collagen matrices have also been utilized to encapsulate MSCs-EVs, enhancing their stability and promoting a continuous release profile, which ameliorates AKI [240]. These innovative approaches not only enhance the therapeutic efficacy of EVs but also pave new avenues for AKI management (Fig. 4b).

### 5.3 Targeting and delivery precision

EVs are renowned for their innate ability to home in on target cells over long distances, a trait that positions them as powerful tools with significant clinical potential. Yet, the targeting prowess of unaltered EVs falls short in clinical settings. To surmount this limitation, scientists are delving into bioengineering strategies to meticulously engineer EVs, aiming to sharpen their precision in homing to specific cells and tissues [241,242]. A Phase 1a clinical trial indicated that the direct infusion of autologous AMSCs via the renal artery can elevate GFR and renal blood flow in patients with atherosclerotic nephropathy, concurrently dampening local inflammatory responses [243]. In a cisplatin-induced AKI mouse model, the direct renal arterial injection of MSCs-derived EVs restored impaired renal function [244]. Despite the inherent risks and technical challenges of direct renal arterial EVs infusion, the expression of specific biomarkers by injured renal tissues provides an avenue for EVs modification to improve therapeutic targeting [245]. For example, in an AKI mouse model, damaged TECs express the KIM-1, prompting researchers to engineer EVs with the KIM-1 binding peptide LTH (LTHVVWL) to enhance their homing to injured renal tissues, thereby effectively mitigating tubular interstitial inflammation and fibrosis [150,246]. Considering the pivotal role of endothelial cells in AKI pathogenesis, researchers have explored strategies to modify EVs for targeting renal vascular endothelium. In AKI triggered by diverse etiologies, P-selectin is highly expressed in glomeruli and peritubular capillaries, facilitating neutrophil infiltration and renal damage. Leveraging hPMSCs-EVs, investigators have developed novel therapeutic EVs equipped with peptides that bind P-selectin (CDAEWVDVS), enhancing their targeted therapeutic efficacy [247,248]. Beyond overexpressing specific homing molecules, studies have also engineered hybrid vesicles (NEX) by integrating human neutrophil membranes with hucMSCs-EVs. These NEX exhibit features of human neutrophil membrane surface proteins, demonstrating superior targeting of damaged renal tissues in a cisplatin-induced AKI mouse model. *In vitro* studies confirm that NEX are preferentially internalized by NRK52E cells over RAW264.7 cells, underscoring their potential for AKI treatment [249] (Fig. 4c).

### 5.4 Therapeutic potency augmentation

Scientists are deploying a suite of strategies to boost the therapeutic efficacy of EVs. In the realm of AKI treatment, two prominent approaches have emerged: genetic modification of parental cells and preculture of parental cells coupled with external environmental stimulation. To amplify the therapeutic potency of MSCs-EVs in AKI, researchers have explored strategies of genetic engineering for the overexpression of salutary molecules. For example, overexpression of the transcription factor Oct-4 in MSCs-EVs, which modulates the Snail gene to curb epithelial-mesenchymal transition, has been shown to markedly enhance treatment efficacy. This is primarily achieved by inhibiting renal fibrosis progression and preventing the evolution of AKI into CKD [250]. MSCs-EVs engineered with glial cell line-derived neurotrophic factor (GDNF) stimulate the SIRT1/eNOS pathway in peritubular capillary endothelial cells, thereby promoting angiogenesis and curbing tubular interstitial fibrosis [251]. Additionally, the overexpression of angiotensin-converting enzyme 2 (ACE2) in MSCs via lentiviral vectors enriches EVs with ACE2. These EVs target damaged kidneys, activate the Nrf 2/HO-1 signaling pathway, inhibit TECs apoptosis, modulate inflammatory responses, and mitigate oxidative stress in IR-AKI [252]. Indoleamine 2,3-dioxygenase (IDO), an immunosuppressive enzyme critical for immune balance, is overexpressed in MSCs-EVs to bolster treatment of IR-AKI. This enhancement involves promoting the phenotypic switch of M1 macrophages to M2, ameliorating the inflammatory milieu within the impaired renal tissue, and fostering tissue repair [58]. Similarly, Anti-inflammatory molecules, including IL-10 and IκBα, have been integrated into EVs engineering. Macrophages transfected with IL-10-expressing plasmids generate EVs rich in IL-10, which target damaged TECs via ITGαLβ2-ICAM-1 interactions, promoting autophagy and anti-inflammatory benefits in IR-AKI [253]. The EXPLOR technique has yielded EVs carrying IκBα, which, upon uptake by neutrophils and macrophages in IR-AKI, inhibit NF-κB activity and exhibit anti-inflammatory properties [254,255]. MicroRNAs, integral to EVs composition, are being scrutinized for low levels in AKI patients or animal models. Identified miRNAs through the analysis are then introduced into MSCs, enabling the production of specific miRNA-enriched EVs. For instance, miR-223–3p, by modulating HDAC2 to enhance SNRK transcription, has demonstrated renal function improvement effects in AKI models, with MSCs-EVs showing potent therapeutic efficacy even at low doses due to the overexpression of specific miRNAs [256,257]. Furthermore, Klotho, a protein identified in urinary EVs that fosters renal recovery, when incorporated into fibroblast-derived EVs, confers renoprotective capabilities [258].

Notably, in a cisplatin-induced AKI mouse model, pulsed focused ultrasound (pFUS) has been shown to markedly enhance the efficacy of MSCs-EVs. Specifically, the synergistic use of pFUS with MSCs-EVs triggers cell proliferation pathways, including MAPK/ERK and PI3K/Akt, and upregulates regenerative markers such as eNOS and SIRT3 [259]. Concurrently, it mitigates inflammation by reducing heat shock protein 70 (HSP70) levels and dampening NLRP3 inflammasome activation [260]. Moreover, MSCs-EVs following hypoxic preconditioning [261] or treatment with pan PPAR agonists [262] display enhanced profiles in anti-inflammatory, anti-apoptotic, and antioxidant actions, as well as in promoting vasculogenesis. These enhancements are likely attributed to the hypoxia-induced strengthening of MSCs immunomodulatory properties and the agonists' inherent ability to foster tissue repair (Fig. 4d).

## 6 Discussion

The mechanisms underlying AKI are intricate, with a multitude of biological and cellular events implicated in the transition from kidney injury to CKD [7,263]. Throughout this process, various cell types, including TECs [264,265], immune cells [266], endothelial cells [267], fibroblasts [268], and podocytes [269], play crucial roles. They modulate critical processes such as inflammation, apoptosis, tubular regeneration, and fibrosis through intricate interactions and signaling pathways. In this complex biological context, intercellular communication is paramount, with EVs serving as pivotal messengers in AKI injury and the subsequent renal fibrosis and repair processes. Despite the growing importance of EVs, research on their role in AKI and the shift to CKD remains scarce. The majority of studies have concentrated on the impact of EVs released by injured TECs post-AKI on their counterparts [25,28,39]. However, emerging evidence points to a significant regulatory function of EVs from other cell sources in AKI progression, particularly immune cells. Single-cell sequencing and bioinformatics analyses of ligand-receptor interactions between immune cells and TECs at various time points post-AKI (4 h, 12 h, 2 days, 14 days, 6 weeks) reveal weak direct effects [270], suggesting that EVs may facilitate communication between these cell types. Further studies support this hypothesis. For instance, macrophage-derived EVs post-AKI, carrying miR-155, act on TECs to promote DNA damage, apoptosis, and inhibit proliferation, exacerbating tissue inflammation and kidney injury [[132], [133], [134]]. Additionally, miR-195a-5p intensifies cell damage and advances AKI by inducing mitochondrial dysfunction in TECs [135]. These insights underscore the critical role of EVs in cell-to-cell communication, especially in the regulation of AKI progression. Future research should concentrate on the intercellular communication mediated by EVs during the transition from AKI to CKD, investigating their role in modulating AKI progression and renal fibrosis or repair. Moreover, while most studies have focused on the RNA content of EVs, there is a pressing need for more research on non-RNA components to uncover their potential contributions to AKI and subsequent fibrosis.

EVs are emerging as a promising treatment for AKI, drawing significant interest. EVs from stem and progenitor cells, such as BMSCs, hucMSCs, and EPCs, have demonstrated vast potential in treating AKI, alleviating kidney damage, and promoting repair and anti-inflammatory effects. However, the progress in therapeutic EVs research and their clinical translation face critical challenges that require further exploration and resolution. Firstly, current studies often concentrate on EVs from a single cell source, lacking comparative analysis, which hinders a thorough evaluation of their therapeutic potential. Comparing the efficacy of EVs from various sources is crucial for both scientific and clinical purposes, helping to pinpoint the most effective therapeutic origins. For example, AMSCs-sEVs outperform BMSCs-sEVs in sepsis-related AKI [101], while hucMSCs-EVs are superior in enhancing TECs' energy metabolism [102]. EVs from different sources may vary in therapeutic outcomes due to differences in biological properties and molecular content. Thus, choosing the right EVs source is vital for treatment efficacy [271,272]. In comparative studies, factors such as EVs dosage, AKI etiology, extraction methods, and operational errors must be considered, as they can impact the consistency and comparability of research findings and should be meticulously addressed. Secondly, despite the potential of EVs from multiple cell sources, clinical application presents challenges. When selecting EVs sources, cost, extraction complexity, and ethical considerations are paramount. There are significant differences in extraction costs and difficulties among EVs from various sources. BMSCs-EVs extraction has been standardized [273], but some specialized EVs extraction can be expensive and intricate. Ethical issues, particularly with embryonic or placental cells, become constraints for clinical use. Future research should holistically consider these factors to devise more feasible clinical application strategies. Lastly, the distinctiveness and complexity of EVs from different cell sources in AKI treatment are intriguing: a. They show specific target and functional preferences; b. Even with the same effector molecules, EVs from different sources may operate through similar or distinct pathways; c. There may be potency differences between sEVs and lEVs from the same cell source.

Despite the promising research on EVs for diagnosing AKI, their clinical application remains unrealized. Key obstacles include the following: Firstly, challenges of isolation and purification, where rapid, simple, stable, and high-recovery methods are lacking, particularly when dealing with plasma samples and the interference from lipoproteins. Secondly, standardization and quality control are also critical issues. Large-scale EVs production requires adherence to strict manufacturing standards and consistent quality and functionality, which are currently hampered by the absence of unified processes. Thirdly, the sensitivity and specificity of EVs-carried biomarkers, such as miRNA and proteins, are promising for AKI diagnosis but require enhancement to ensure diagnostic precision, as varying clinical contexts can affect their performance. Lastly, the transition from lab to clinic is complex, necessitating technological advancements in high-throughput detection and standardization across laboratories. Current technologies have yet to meet clinical standards, thus limiting EVs' practical application in AKI diagnosis. Overcoming these challenges is essential for future research advancements [274,275].

The engineering modification of EVs holds significant promise for the treatment of AKI [226]. The key challenges involve improving EVs' yield, purification efficiency, circulation stability, targeting ability, and therapeutic efficacy. These topics have been discussed in previous sections. Additionally, we must pay attention to emerging technologies, particularly the development of *in vivo* tracking methods for EVs. Tracking the distribution of MSCs-EVs in real-time is essential for refining AKI treatment strategies. Scientists have harnessed a magnetic bead-based tracing technique for MRI visualization of EVs within damaged renal tissues, optimizing therapeutic approaches [276]. In preclinical AKI research, a co-assembly system integrating MSCs-EVs with the macrocyclic amphiphile C5A and the hypoxia-sensitive dye sulfonated aluminum phthalocyanine (*Pc*) has been developed. This Pc/C5A complex emits fluorescent signals *in vitro*, indicative of hypoxia, without compromising the biological activity of MSCs-EVs [277]. Additionally, the 3D-MOTIVE Chip has been instrumental in mimicking cisplatin-induced nephrotoxicity, enabling rapid assessment of MSCs-EVs' therapeutic impact on injured TECs and underscoring their potential in mitigating cisplatin-induced renal damage [278]. In parallel, a 3D gravity-driven microfluidic platform has been engineered to replicate acute hypoxic injury in TECs, allowing for precise evaluation of the therapeutic efficacy of exosomes from BMSCs on hypoxia-induced AKI, based on cellular structural and functional alterations [279]. The deployment of these cutting-edge methodologies is poised to accelerate preclinical advancements in AKI treatment utilizing EVs. Furthermore, exploring the synergistic application of various engineering technologies is also a promising research direction. For example, dexamethasone (DXM), which is widely utilized in renal therapy, poses significant side effects with chronic, high-dose use, underscoring the importance of developing targeted dexamethasone therapies. Studies have demonstrated that EVs from macrophages cultured in dexamethasone can transport the drug, targeting inflamed renal environments via integrins LFA-1 and VLA-4, thereby exerting anti-inflammatory effects [280]. The encapsulation of IL-37 protein, a novel anti-inflammatory molecule with inherent instability and delivery challenges [281], within neutrophil membrane-derived EVs has been achieved, creating stable nanosized vesicles. These vesicles harness PSGL-1 to target damaged endothelial cells, releasing IL-37 to stimulate endothelial proliferation, angiogenesis, and prevent inflammatory cell infiltration, thus alleviating AKI [282].

As insights into the mechanisms of EVs action in AKI grow, and their potential as diagnostic and therapeutic agents is increasingly recognized, EVs research is becoming a cornerstone in nephrology. While challenges in EVs isolation, enrichment, and clinical translation remain, the advent of innovative technologies is steadily surmounting these barriers. The future of AKI management looks poised to integrate the refined engineering of EVs with precision medicine, positioning them as a formidable tool for diagnostics and therapeutics. This progress is expected to enrich treatment options for AKI, invigorate the fields of regenerative and nanomedicine, and lead to more timely diagnoses, precise interventions, and holistic disease management. With the relentless advancement of EVs research, there is considerable optimism that these vesicles will assume a pivotal role in AKI therapy, bringing new avenues of hope to patients.

## CRediT authorship contribution statement

**Sirui Li:** Resources, Project administration, Conceptualization. **Lan Zhou:** Writing – review & editing, Supervision, Funding acquisition, Conceptualization. **Yu Huang:** Writing – review & editing, Visualization, Conceptualization. **Shupei Tang:** Writing – review & editing, Writing – original draft, Funding acquisition, Conceptualization.

## Financial Support and sponsorship

Sirui Li and Lan Zhou contribute equally. This work is financially supported by the 10.13039/501100001809National Natural Science Foundation of China (No.82400812 and No.82403293) and the Natural Science Foundation of Shigatse City (No. RKZ2024ZR-002).

## Declaration of competing interest

The authors declare no conflict of interest.
